# Optimisation of the Synthesis and Cell Labelling Conditions for [^89^Zr]Zr-oxine and [^89^Zr]Zr-DFO-NCS: a Direct *In Vitro* Comparison in Cell Types with Distinct Therapeutic Applications

**DOI:** 10.1007/s11307-021-01622-z

**Published:** 2021-07-06

**Authors:** Ida Friberger, Emma Jussing, Jinming Han, Jeroen A. C. M. Goos, Jonathan Siikanen, Helen Kaipe, Mélanie Lambert, Robert A. Harris, Erik Samén, Mattias Carlsten, Staffan Holmin, Thuy A. Tran

**Affiliations:** 1 Department of Clinical Neuroscience, Karolinska Institutet, Stockholm, Sweden; 2 Department of Oncology and Pathology, Karolinska Institutet, Stockholm, Sweden; 3 Department of Radiopharmacy, Karolinska University Hospital, Stockholm, Sweden; 4 Centre for Molecular Medicine, Karolinska University Hospital, Stockholm, Sweden; 5 Department of Medical Radiation Physics and Nuclear Medicine, Karolinska University Hospital, Stockholm, Sweden; 6 Department of Laboratory Medicine, Karolinska Institutet, Stockholm, Sweden; 7 Department of Clinical Immunology and Transfusion Medicine, Karolinska University Hospital, Stockholm, Sweden; 8 Department of Medicine in Huddinge, Karolinska Institutet, Stockholm, Sweden; 9 Center for Cell Therapy and Allogeneic Stem Cell Transplantation (CAST), Karolinska University Hospital, Stockholm, Sweden; 10 Department of Neuroradiology, Karolinska University Hospital, Stockholm, Sweden

**Keywords:** Cell labelling, Cell tracking, ^89^Zr, Oxine, Deferoxamine, PET, Imaging

## Abstract

**Background:**

There is a need to better characterise cell-based therapies in preclinical models to help facilitate their translation to humans. Long-term high-resolution tracking of the cells *in vivo* is often impossible due to unreliable methods. Radiolabelling of cells has the advantage of being able to reveal cellular kinetics *in vivo* over time. This study aimed to optimise the synthesis of the radiotracers [^89^Zr]Zr-oxine (8-hydroxyquinoline) and [^89^Zr]Zr-DFO-NCS (p-SCN-Bn-Deferoxamine) and to perform a direct comparison of the cell labelling efficiency using these radiotracers.

**Procedures:**

Several parameters, such as buffers, pH, labelling time and temperature, were investigated to optimise the synthesis of [^89^Zr]Zr-oxine and [^89^Zr]Zr-DFO-NCS in order to reach a radiochemical conversion (RCC) of >95 % without purification. Radio-instant thin-layer chromatography (iTLC) and radio high-performance liquid chromatography (radio-HPLC) were used to determine the RCC. Cells were labelled with [^89^Zr]Zr-oxine or [^89^Zr]Zr-DFO-NCS. The cellular retention of ^89^Zr and the labelling impact was determined by analysing the cellular functions, such as viability, proliferation, phagocytotic ability and phenotypic immunostaining.

**Results:**

The optimised synthesis of [^89^Zr]Zr-oxine and [^89^Zr]Zr-DFO-NCS resulted in straightforward protocols not requiring additional purification. [^89^Zr]Zr-oxine and [^89^Zr]Zr-DFO-NCS were synthesised with an average RCC of 98.4 % (n = 16) and 98.0 % (n = 13), respectively. Cell labelling efficiencies were 63.9 % (n = 35) and 70.2 % (n = 30), respectively. ^89^Zr labelling neither significantly affected the cell viability (cell viability loss was in the range of 1–8 % compared to its corresponding non-labelled cells, *P* value > 0.05) nor the cells’ proliferation rate. The phenotype of human decidual stromal cells (hDSC) and phagocytic function of rat bone-marrow-derived macrophages (rMac) was somewhat affected by radiolabelling.

**Conclusions:**

Our study demonstrates that [^89^Zr]Zr-oxine and [^89^Zr]Zr-DFO-NCS are equally effective in cell labelling. However, [^89^Zr]Zr-oxine was superior to [^89^Zr]Zr-DFO-NCS with regard to long-term stability, cellular retention, minimal variation between cell types and cell labelling efficiency.

**Supplementary Information:**

The online version contains supplementary material available at 10.1007/s11307-021-01622-z.

## Background

Novel cell therapies are constantly emerging as promising treatments for a variety of cancers. Successful examples include the use of chimeric antigen receptor (CAR) T cells to treat patients with acute lymphoid leukemia, lymphoma and multiple myeloma and natural killer cells for acute myeloid leukemia, as well as the treatment of post-transplant leukemia relapse with donor lymphocyte infusions (DLIs) [1–6]. Furthermore, stem cell transplantation has proven successful in the treatment of hematological malignancies, autoimmune diseases, immune deficiencies, metabolic syndromes and the regeneration of tissues [4, 7–10]. However, several of these cell therapies remain unpredictable in terms of efficacy and potential side effects. A change of strategies is required in order to improve efficacy while minimising the risk of complications [11–13]. With the rapid increase of new and more targeted cell therapies, it is essential to develop reliable tools to better understand the biodistribution of the cell therapy product once infused *in vivo* [12, 13]. Three-dimensional, long-term tracking of radiolabelled cells using positron emission tomography (PET), particularly in combination with computed tomography (CT) or magnetic resonance imaging (MRI), could provide this important information [14].

The most commonly used radiotracers for cell tracking in clinical practice are the single-photon emission computed tomography (SPECT) tracers [^111^In]In-oxine and [^99m^Tc]Tc-HMPAO, which have been applied for the radiolabelling of leucocytes to detect infections and inflammation [14–17].

Compared to SPECT, PET imaging provides higher spatial resolution, better sensitivity and quantification ability [18]. PET tracers that have been used for cell labelling include [^18^F]FDG [17, 19], [^64^Cu]Cu-PTSM, [^124^I]FIAU [20], [^124^I]FIT-Mal and [^124^I]FIT-(PhS)2-Mal [21]. Unfortunately, these tracers have either too short half-lives, which are not suitable for long-term cell tracking, and/or low cellular retention, causing a high radioactive dose to the blood, bone marrow and excretion organs, and a low target-to-background ratio.

**Fig. 1.**
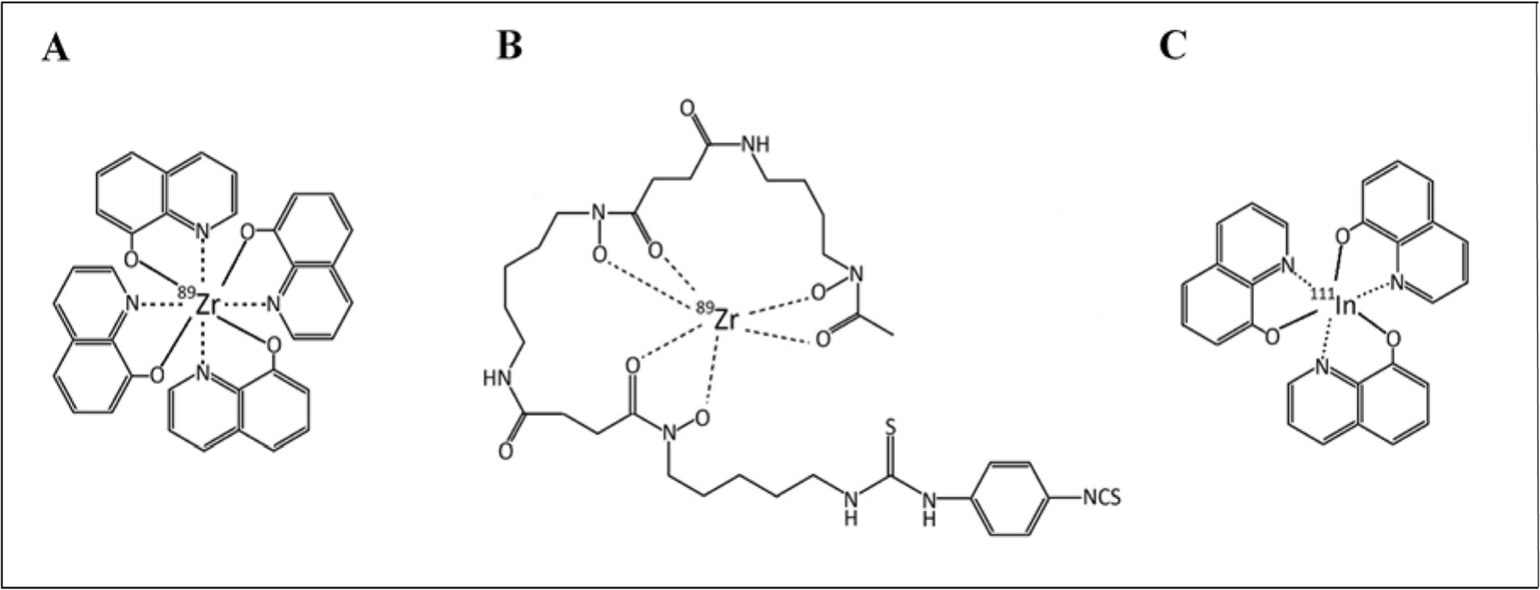
Molecular structures of ^89^Zr-based PET radiotracers **A** [^89^Zr]Zr-oxine and **B** [^89^Zr]Zr-DFO-NCS, and the previously established SPECT radiotracer **C** [^111^In]In-oxine.

The possibility to perform long-term cell tracking using zirconium-89 (^89^Zr) is attractive and feasible. ^89^Zr has a half-life of 3.3 days and emits positrons with a mean energy of 395 keV, which enables high-resolution PET imaging for up to 3 weeks [14]. Recent developments in cell labelling strategies using ^89^Zr-complexed 8-hydroxyquinoline ([^89^Zr]Zr-oxine) and deferoxamine ([^89^Zr]Zr-DFO-NCS) exhibit great promise. A clinical trial is currently ongoing to investigate the potential of [^89^Zr]Zr-oxine to track lymphocytes and their capability to enter the central nervous system [22]. The advantage of [^89^Zr]Zr-oxine over other PET tracers is its similarity to the clinically established SPECT tracer [^111^In]In-oxine (Fig. 1), which may help facilitate clinical translation. The currently available protocols for [^89^Zr]Zr-oxine synthesis are characterised by a low overall radiochemical yield (RCY) and include cumbersome steps such as buffer exchange, chloroform extractions, evaporations and the usage of several buffers in the synthesis [16, 23–26].

**Fig. 2.**
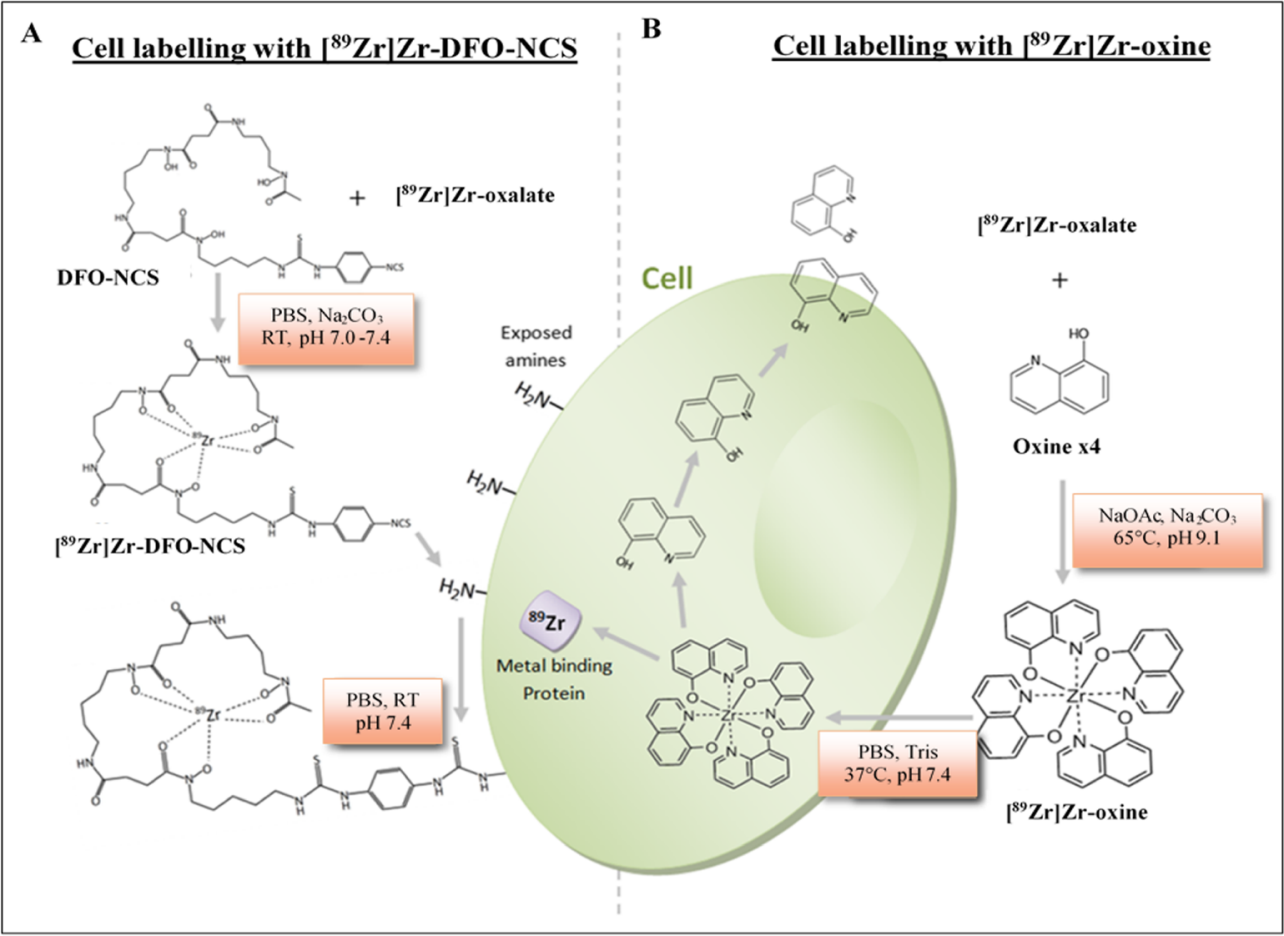
Schematic illustration of the synthesis conditions and cell labelling mechanisms of (A) extracellular labelling using [^89^Zr]Zr-DFO-NCS and (B) intracellular labelling using [^89^Zr]Zr-oxine.

Conversely, the synthesis of [^89^Zr]Zr-DFO-NCS is simpler and has been investigated in several studies since its introduction by Bansal *et al* [14, 27, 28]. The [^89^Zr]Zr-oxine complex forms a hydrophobic sphere, is neutral and lipid soluble, which allows it to passively diffuse across the cell membrane. Once inside the cell, ^89^Zr is intracellularly trapped by unspecific binding to the cytoplasmic components, while the liberated oxine molecules leave the cell [29]. On the other hand, [^89^Zr]Zr-DFO-NCS binds to exposed amines on the cell membrane surface, as illustrated in Fig. 2. Both labelling methods have their advantages and disadvantages but until now there has been no direct comparison of both methods to clarify which is more suitable for long-term cell tracking *in vivo*.

This study aimed to optimise protocols for the synthesis of [^89^Zr]Zr-oxine and [^89^Zr]Zr-DFO-NCS without the need for a post-labelling purification step, and to directly compare of the feasibility and efficacy of labelling cells with either [^89^Zr]Zr-oxine or [^89^Zr]Zr-DFO-NCS for distinct therapeutic applications.

## Materials and Methods

### Production of Zirconium-89

^89^Zr was purchased from PerkinElmer or produced in-house with a cyclotron (PETtrace 800, GE Healthcare) using the ^89^Y(p,n)^89^Zr reaction. In-house production of ^89^Zr is described in details in electronic supplementary material (ESM).

### Optimization of [^89^Zr]Zr-oxine Synthesis

Several parameters were investigated to improve the radiochemical conversion (RCC) from the original protocol [16, 24–26], as well as to minimise the timeframe of radioactive exposure. See supplementary. In short, the final protocol was obtained by mixing aliquots of 5–20 MBq [^89^Zr]Zr-oxalate with 50 μL 0.1 M sodium acetate buffer (NaOAc, pH 2.5–3.0) (Merck Millipore) in a centrifuge tube. Thereafter, 0.1 mg dissolved oxine (in 99 % ethanol) was added to the mixture and the pH was adjusted to 9.1 using 1 M sodium carbonate (Honeywell). The sample was incubated for 60 min at 65 °C. The RCC was determined by instant thin-layer chromatography (iTLC) and was confirmed by radio-HPLC (see details in supplementary).

### Optimization of [^89^Zr]Zr-DFO-NCS Synthesis

To achieve a stable RCC of >95 % without the need for purification, we optimised several conditions such as synthesis buffer, reaction time, DFO-NCS concentration and temperature (see supplementary). The RCC in all tests was determined by iTLC using a mobile phase of 50 mM DTPA (Sigma-Aldrich), pH 4, (see supplementary). After all these optimisations, a final protocol was obtained by adding 4 μg DFO-NCS to a mixture of 5–25 MBq of [^89^Zr]Zr-oxalate and 50 μL 0.5 M PBS buffer (Sigma-Aldrich). The pH was adjusted to 7.0–7.4 using 1 M sodium carbonate. Incubation was performed under mild agitation (300 rpm) for 60 min at room temperature. The RCC was determined by iTLC and was confirmed by radio-HPLC as described in supplementary.

### Cell Preparations and Radiolabelling

Human decidual stromal cells (hDSCs), rat bone marrow–derived macrophages (rMac) and human peripheral blood mononuclear cells (hPBMCs) were isolated, cultured and radiolabelled with [^89^Zr]Zr-oxine or [^89^Zr]Zr-DFO-NCS. Cell labelling efficiency (CLE) and cell viability was determined as described in supplementary.

### Cellular Retention and Proliferation

Cellular retention of the radioactivity was measured to estimate leakage of ^89^Zr from the radiolabelled cells. Approximately 1.5–10 × 10^6^ radiolabelled cells were cultured in either complete DMEM or RPMI 1640 medium (10 % FBS and 1 % streptomycin). The culture media was refreshed after 1 and 3 days and the discarded media were stored for gamma counting (Wallac Wizard 1480, Perkin Elmer). After 7 days, cells and supernatant were collected and analysed for retained radioactivity, cell count, and viability.

### Flow Cytometry Analysis

#### Rat Bone Marrow-Derived Macrophages

The phagocytic ability of macrophages was determined using Dextran Alexa Fluor™ 647 (Thermo Fisher) and analysed by flow cytometry (Guava 12HT®, 640-nm laser, Merck Millipore). Dextran in PBS (5 mg/mL) was added to a suspension of macrophages in 1 mL 0.5 M PBS. Cells were incubated at 37 °C for 30 min and then centrifuged (5 min, 1000 rpm). Subsequently, the supernatant was removed and the pellet resuspended in 1 mL 0.5 M PBS to allow for analysis using the Merck Guava easyCyt system 12TH.

#### Human Decidual Stromal Cells

To assess potential alterations in membrane receptor expression caused by radiolabelling procedures, repeated measurements of key antigens were performed using flow cytometry. A fluorochrome panel of anti-human monoclonal antibodies showing positive expression of CD29 (MAR4) (BD), CD44 (IM7), CD73 (AD2), CD105 (SN6) and CD14 (61D3) (Thermo Fisher) as well as CD90 (5E10) (BD) and negative expression of CD34 (4H11) and CD45 (HI30) (Thermo Fisher) would confirm a preserved phenotype of hDSCs. Cells were stained with an antibody concentration of 5 μL/10^6^ cells in 100 μL 0.5 M PBS and incubated for 30 min at 4 °C. Cells were then centrifuged and washed with PBS before flow cytometric analysis.

### Statistical Analysis

Data were decay-corrected and presented as average values with their standard deviation (SD). *P* values were calculated using the Student’s t test in Excel office (Microsoft office pro. plus 2019, version 1808) and *P* < 0.05 was considered as being statistically significant.

## Results

### Optimisation of [^89^Zr]Zr-oxine and [^89^Zr]Zr-DFO-NCS Synthesis

In our initial protocol, a chloroform-based extraction was used to separate the [^89^Zr]Zr-oxine from the unbound ^89^Zr. This protocol resulted in a loss of more than 40 % ^89^Zr before evaporation and an additional 10–30 % was lost when dissolving the dried [^89^Zr]Zr-oxine pellet in DMSO (see Table 1).

**Table 1.** Optimization of [^89^Zr]Zr-oxine and [^89^Zr]Zr-DFO-NCS synthesis

[^89^Zr]Zr-oxine		[^89^Zr]Zr-DFO-NCS	
Conditions	RCC	Conditions	RCC
Chloroform, H_2_O 3.4 mM (0.5 mg) oxine, RT, pH 7.0, 30 min	58.3 % (9.8 SD) n = 8	HEPES and Tris buffers 30 μM DFO-NCS 37 °C, pH 7.4, 30 min	92.4 % (4.9 SD) n = 5
Final protocol: NaOAc, 9.6 mM (0.1 mg) oxine 65 °C, pH 9.1, 60 min	98.4 % (1.3 SD) n = 15	Final protocol: PBS, 70 μM DFO-NCS RT, pH 7.4, 60 min	98.0 % (0.6 SD) n = 13
Scaled-up final synthesis 52–71 MBq	98.7 % (0.4 SD) n = 4	Scaled-up final synthesis 36-45 MBq	97.5 % (0.1 SD) n = 3
Shelf-life CLE	≤ 7 days 60.9 % (4.2 SD) n = 4	Shelf-life CLE	≤ 1.5 h 69.7 % (8.0 SD) n = 4

Steps towards optimization of [^89^Zr]Zr-oxine and [^89^Zr]Zr-DFO-NCS synthesis. Optimization of buffer, temperature and pH resulted in an improved radiochemical conversion (RCC) > 95 % in the final protocols; therefore, no further purification was needed. Cell labelling efficiency (CLE) of rMac, hDSCs or hPBMCs is equally feasible using either freshly synthesised or 7-day-old [^89^Zr]Zr-oxine, while the shelf-life of [^89^Zr]Zr-DFO-NCS is limited to within less than 2 h

**Fig. 3.**
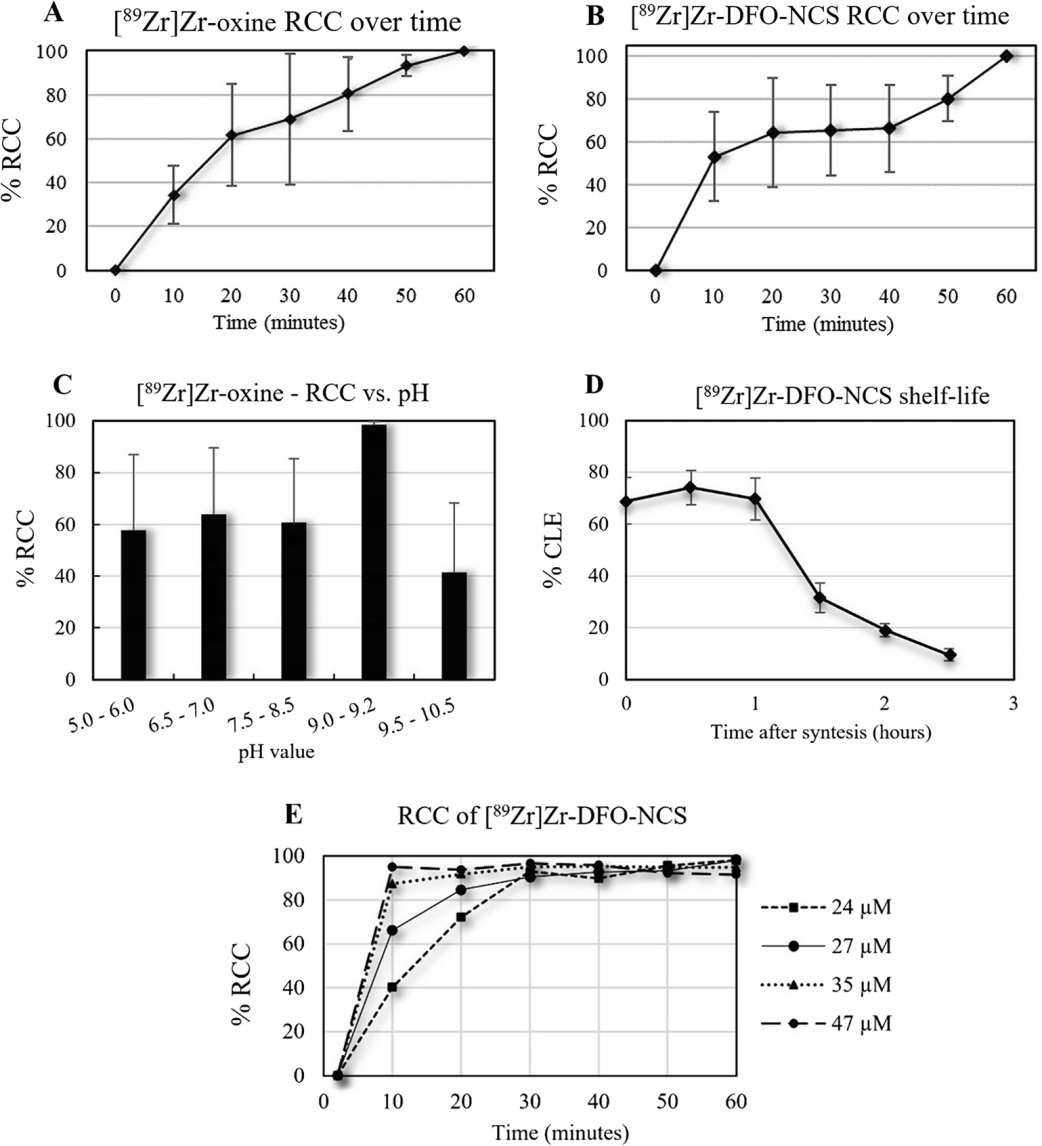
The radiochemical conversion (RCC) during synthesis over time for **A** [^89^Zr]Zr-oxine with an oxine concentration of 7.7 mM at 65 °C and **B** [^89^Zr]Zr-DFO-NCS at a concentration of 7.5 μM in room temperature. Both radiotracers reach an RCC > 98 % after 60 min incubation, and **C** pH-dependent RCC in the synthesis of [^89^Zr]Zr-oxine. **D** Shelf-life of [^89^Zr]Zr-DFO-NCS in pH 7.4 for optimal cell labelling efficiency (CLE). The maximum CLE was obtainable within 1-h post-synthesis. **E** Increased RCC of [^89^Zr]Zr-DFO-NCS with increased DFO-NCS concentration over time.

**Fig. 4.**
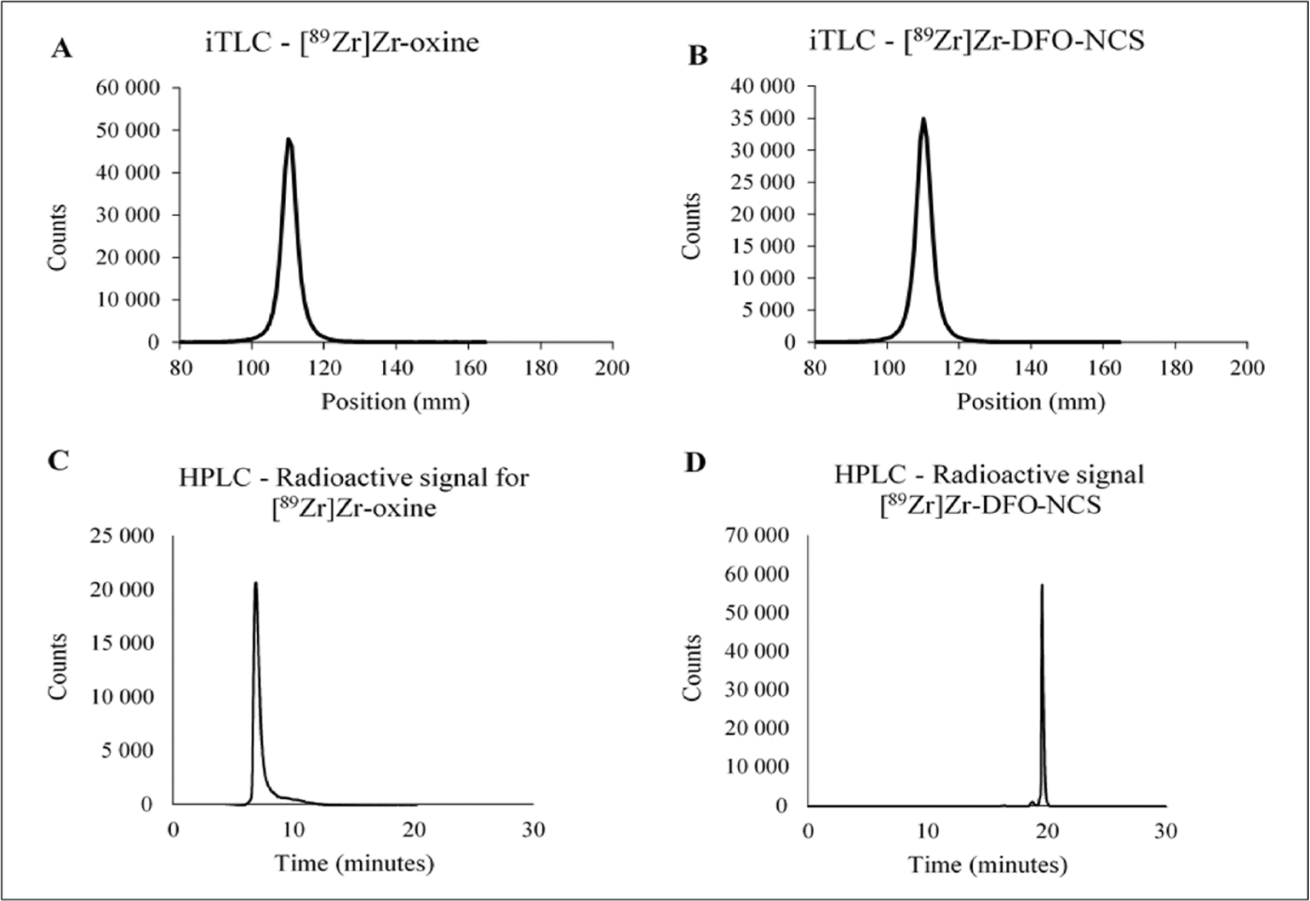
Radiochemical conversion (RCC) >98 % with instant thin-layer chromatography (iTLC) for **A** [^89^Zr]Zr-oxine and **B** [^89^Zr]Zr-DFO-NCS synthesis. Radio-HPLC showing the **C** radioactive peak for [^89^Zr]Zr-oxine and the **D** radioactive peak for [^89^Zr]Zr-DFO-NCS.

To optimise the protocol, we initially changed the chloroform to NaOAc buffer and increased the temperature to 50 °C in a pH range of 5–8. The resulting average RCC was low (>10 %) but showed a higher yield for higher pH > 6. Secondly, we repeated this experiment at a temperature of 65 °C in a pH range of 5–10.5. The highest RCC of 81.2 % (11.2 SD) (n = 7) was obtained at a pH of 9.0–9.2 after 30-min incubation at 65 °C. To avoid the need for purification, we increased the incubation time further and found that the RCC reached 98.4 % (1.3 SD) (n = 16) with 9.6 mM (2.4 SD) oxine after 60 min (Table 1) and (Fig. 3A). As a result, our final protocol describes a total 80-min procedure, with an RCC of > 98 %, as confirmed by iTLC and HPLC (Fig. 4A, C). An important advantage of our protocol is that a further purification step, such as chloroform-based extraction, is not needed. The shelf-life of [^89^Zr]Zr-oxine after 1 week at room temperature in PBS (pH 7.4) was tested and no decrease in purity 98.4 % (0.1 SD) (*P* = 0.4) or change in cell labelling capability 60.9 % (4.2 SD) (Table 1) was apparent.

The optimisation of the [^89^Zr]Zr-DFO-NCS synthesis was significantly less time-consuming. In the initial protocol, 12 μg (35 μM) DFO-NCS was incubated at 37 °C for 30 min in a pH 7.4 solution of 250 μL HEPES buffer, 200 μL Tris buffer, and 50–100 μL [^89^Zr]Zr-oxalate. This protocol yielded an RCC of 92.4 % (4.9 SD) (n = 5). We found no significant difference in RCC between HEPES and PBS as well as the presence of Tris buffer. The final procedure was therefore performed in PBS pH 7.4, without Tris buffer. By decreasing the volume and increasing the amount of DFO-NCS, an RCC of >95 % was achieved after approximately 10 min of incubation (Fig. 3E). Unfortunately, we found that DFO-NCS had a toxic effect on cells when exceeding a cell labelling concentration of approximately 9 μM (6 μg). The cell labelling concentration of [^89^Zr]Zr-DFO-NCS was therefore kept below 8 μM, which corresponds to a synthesis concentration of 27 μM. Another attempt to accelerate the reaction was to increase the temperature to 95 °C, which substantially decreased the RCC to 69.7 % (9.1 SD) (n = 3). To achieve a stable RCC of >95 % at room temperature, we therefore increased the incubation time to 60 min (Fig. 3B). This provided an RCC of 98.0 % (0.6 SD) (n = 13), as confirmed by iTLC and HPLC (Fig. 4B, D).

Both protocols for [^89^Zr]Zr-oxine and [^89^Zr]Zr-DFO-NCS have been scaled up to a clinically relevant production level without affecting the RCC (Table 1).

### [^89^Zr]Zr-oxine Labelling of hDSCs, rMac and hPBMC

All cells were successfully labelled with [^89^Zr]Zr-oxine. Within 40 min at 37 °C, the CLE was 61.0 % (6.2 SD) (n = 11) for hDSCs and 62.2 % (10.6 SD) (n = 10) for rMac (Table 2), respectively. The loss in cell viability was 2.0 % (2.9 SD) (n = 11, *P* = 0.19) for hDSC, 4.5 % (3.5 SD) (n = 10, *P* = 0.13) for rMac and 7.9 % (14.8 SD) (n = 14, *P* = 0.45) for hPBMC (Table 2). After a 7-day shelf-life in PBS (pH 7.4) at room temperature, the cell labelling capacity of [^89^Zr]Zr-oxine appeared unaltered, with a CLE of 60.9 % (4.2 SD) (n = 4) for hDSCs (Table 1). There were no significant decreases in cell viability before and after cell labelling.

**Table 2.** Radiolabelling of human decidual stromal cells (hDSCs), rat macrophages (rMac) and human peripheral blood mononuclear cells (hPBMC)

	[^89^Zr]Zr-oxine			[^89^Zr]Zr-DFO-NCS			[^111^In]In-oxine		
	hDSCs	rMac	hPBMC	hDSCs	rMac	hPBMC	hDSCs	hMac	hPBMC
CLE	61.0 %	62.2 %	68.6 %	71.3 %	68.9 %	70.2 %	76.0 %*	65.2 %**	71 %***
	6.2 S	10.6 SD	9.9 SD	7.2 SD	12.7 SD	13.5 SD	8.0 SD	0.9 SD	13.9 SD
Samples	n = 11	n = 10	n = 14	n = 12	n = 9	n = 9	n = 6	n = 13	n = 8
Bq/cell	5.3 Bq	4.3 Bq	2.4 Bq	4.5 Bq	4.5 Bq	1.0 Bq	1.8 Bq*	3.6 Bq**	2.2 Bq
	1.8 SD	6.8 SD	2.0 SD	2.7 SD	2.5 SD	0.8 SD	0.4 SD	2.1 SD	1.4 SD
Cell viability loss	2.0 %	4.5 %	7.9 %	1.4 %	4.0 %	1.1 %	NA^x^	4.7 %**	~1 %
	2.9 SD	3.5 SD	14.8 SD	4.0 SD	5.8 SD	6.6 SD		7.6 SD	NA SD
*P-*value	0.19	0.13	0.45	0.08	0.06	0.6	NA	0.33	NA

Radiolabelling of human decidual stromal cells (hDSCs), rat macrophages (rMac) and human peripheral blood mononuclear cells (hPBMC) using [^89^Zr]Zr-oxine and [^89^Zr]Zr-DFO-NCS, in comparison to commercially available [^111^In]In-oxine [29]. There was no significant difference in cell labelling efficiency (CLE) between the radiotracers regarding rMac and hPBMC. Previous studies on [^111^In]In-oxine still propose a superior labelling yield for hDSCs compared to [^89^Zr]Zr-oxine [29, 35]. None of the radiotracers showed any significant effect on the cellular viability with a dosage of 1.0–5.3 Bq/cell. *Arnberg et al. [29], **Lundberg et al. to be submitted 2021, ***Weiner et al. [45]. ^x^Not applicable for hDSCs.

### [^89^Zr]Zr-DFO-NCS Labelling of hDSCs, rMac and hPBMC

Viability tests on hDSC labelled with [^89^Zr]Zr-DFO-NCS showed a decrease in cell viability with increasing concentration of [^89^Zr]Zr-DFO-NCS. Labelling cells with a concentration of 7.5 μM (2.3 SD) [^89^Zr]Zr-DFO-NCS resulted in CLEs of 71.3 % (7.2 SD) (n = 12) for hDSCs, 68.9 % (12.7 SD) (n = 9) for rMac and 70.2 % (13.5 SD) (n = 9) for hPBMC, respectively. No significant decrease in cell viability was observed under this condition (Table 2).

The shelf-life of [^89^Zr]Zr-DFO-NCS at room temperature and pH 7.4 was shorter than that of [^89^Zr]Zr-oxine (<1.5 hrs *vs* 7 days for [^89^Zr]Zr-oxine). The CLE of [^89^Zr]Zr-DFO-NCS decreased from 69 % after a waiting period of 1 h between radiosynthesis and cell labelling to 31 % for a 1.5-hwaiting period, and to less than 10 % when the waiting period exceeded 2.5 h (Fig. 3D).

### Cellular Retention of [^89^Zr]Zr-oxine and [^89^Zr]Zr-DFO-NCS

The retention of [^89^Zr]Zr-oxine and [^89^Zr]Zr-DFO-NCS in the cells was measured *in vitro* at 24 h and 7 days post-labelling. During the first 24 h, there was an apparent loss of retention in all three cell types (Table 3). The cellular retention of [^89^Zr]Zr-oxine at day 7 had dropped to 56–70 %. The 7-day cellular retention of [^89^Zr]Zr-DFO-NCS was 60.3 % (4.9 SD) for hDSC, 25.5 % (1.6 SD) for rMac and 44.0 % (30.3 SD) for hPBMC. This corresponds to a radioactive efflux of 29–38 % for [^89^Zr]Zr-oxine and of 31–60 % for [^89^Zr]Zr-DFO-NCS (Table 3). A control experiment with free ^89^Zr showed that less than 1 % (n = 3) of radioactivity was taken up by the cells.

**Table 3.** Cellular retention

	[^89^Zr]Zr-oxine			[^89^Zr]Zr-DFO-NCS		
	hDSCs	rMac	hPBMC	hDSCs	rMac	hPBMC
Retention 24 h	68.4 %	66.3 %	61.5 %	64.6 %	40.2 %	69.0 %
	13.4 SD	8.3 SD	4.3 SD	14.7 SD	3.3 SD	13.0 SD
Retention 7 days	56.5 %	56.1 %	59.6 %	60.3 %	25.5 %	44.0 %
	21.0 SD	6.0 SD	3.5 SD	4.9 SD	1.6 SD	30.3 SD
Proliferation Labelled cells	1.58	2.25	0.75	1.37	2.31	0.75
	0.06 D	0.40 SD	0.04 SD	0.03 SD	0.24 SD	0.12 SD
Samples	n = 3	n = 5	n = 3	n = 3	n = 3	n = 3
Proliferation Control cells	1.44	2.29	0.81	1.44	2.29	0.81
	0.31 SD	0.14 SD	0.05 SD	0.31 SD	0.14 SD	0.05 SD
Samples	n = 3	n = 3	n = 4	n = 3	n = 3	n = 4
*P* value	0.2	0.4	0.1	0.4	0.5	0.3

Cellular retention of [^89^Zr]Zr-oxine and [^89^Zr]Zr-DFO-NCS in human decidual stem cells (hDSCs), rat macrophages (rMac) and human peripheral blood mononuclear cells (hPBMC) *in vitro*, 24 h and 7 days post labelling. Radiolabelled cells showed no significant decrease in proliferation rate after 7 days of culture compared to controls of unlabelled cells

### Proliferation, Phenotype and Function of Cells After Labelling

The radiolabelling did not have a significant effect on the proliferation rate compared to unlabelled cells (Table 3).

**Fig. 5.**
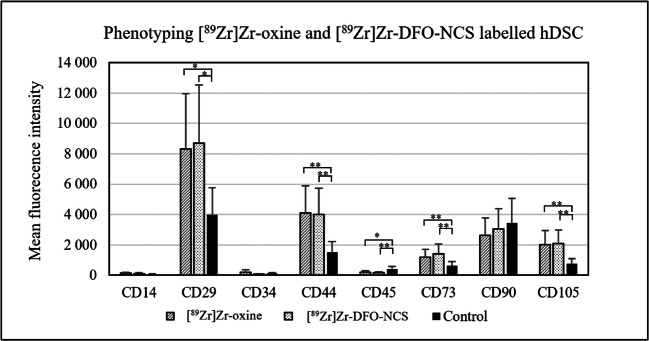
Phenotyping by measuring antigen expression of hDSC pre- and post-labelling with [^89^Zr]Zr-oxine (n = 3) or [^89^Zr]Zr-DFO-NCS (n = 3). A flow panel for positive expression of CD29, CD44, CD73, CD90 and CD105 and a negative expression of CD14, CD34 and CD45 corresponds to hDSC phenotype. Radiolabelled cells showed a significant increase in expression of CD29, CD44, CD73 and CD105 compared to unlabelled cells (n = 3), which still corresponds to hDSC phenotype. Data were analysed as two parametric, paired-samples (pre- vs. post-labelling) with student T-test, *P* values set as <0.05*, < 0.01**. Bars represent mean and error bars standard deviation.

Phenotyping was performed by flow cytometry by measuring antigen expression on hDSCs pre- and post-labelling with [^89^Zr]Zr-oxine and [^89^Zr]Zr-DFO-NCS. No major phenotypic changes were detected in hDSCs after labelling. Since cells were previously isolated and characterised as hDSCs, the phenotype post-labelling was determined using a slightly reduced antibody panel. A positive expression of CD29, CD44, CD73, CD90 and CD105 and a negative expression of CD14, CD34 and CD45 would confirm that radiolabelling did not alter the cellular phenotype. However, radiolabelled hDSCs showed a significant increase in expression of CD29, CD44, CD73 and CD105 compared to unlabelled control cells (Fig. 5).

**Fig. 6.**
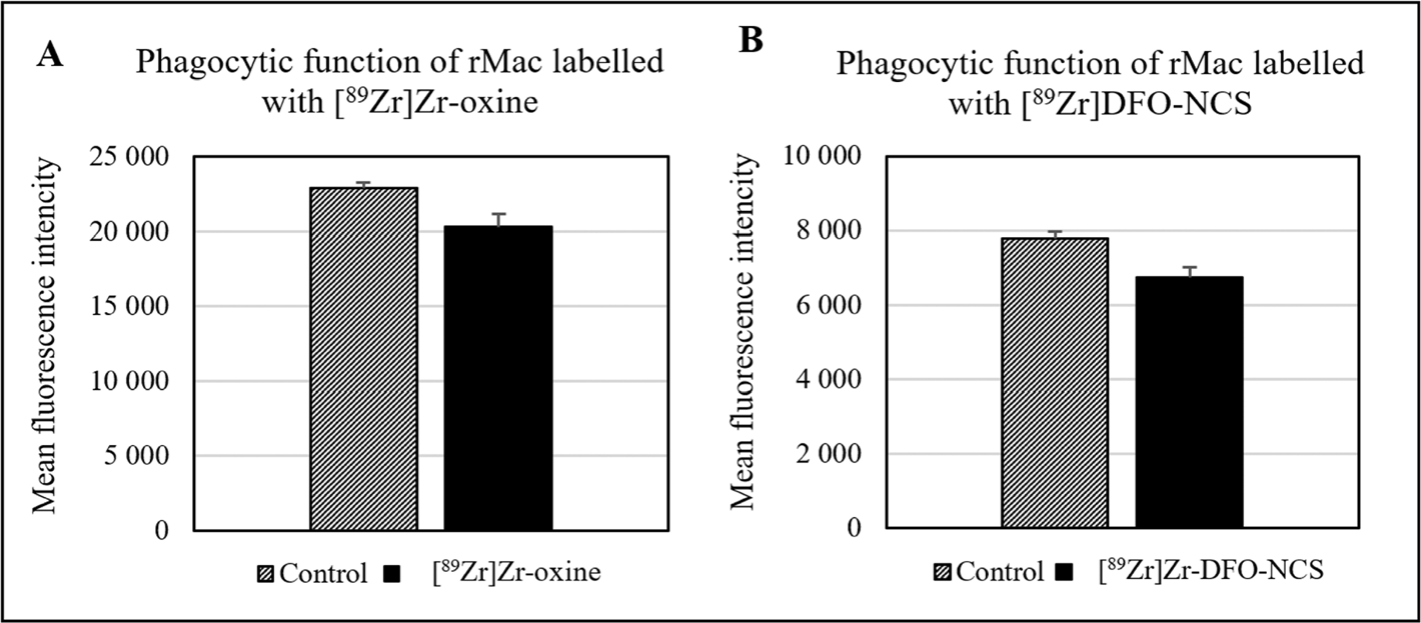
Phagocytic function measured with fluorescent dextran uptake in rat macrophages after labelling with **A** [^89^Zr]Zr-oxine and **B** [^89^Zr]Zr-DFO-NCS. The phagocytotic function shows a slight decrease for both compounds compared to unlabelled cells. [^89^Zr]Zr-oxine labelled cells decrease with 11.2 % (3.6 SD) (n = 3, *P* = 0.048) and [^89^Zr]Zr-DFO-NCS labelled cells decrease with 13.3 % (4.7 SD) (n = 3, *P* = 0.062). Bars represent mean and error bars standard deviation.

Phagocytic function of rMac was measured by assessing fluorescent dextran uptake in cells labelled with either [^89^Zr]Zr-DFO-NCS or [^89^Zr]Zr-oxine. Labelled cells showed a slightly decreased phagocytotic function compared to unlabelled rMac. Dextran uptake in rMac labelled with [^89^Zr]Zr-oxine decreased significantly by 11.2 % (3.6 SD) (n = 3, *P* = 0.048) and cells labelled with [^89^Zr]Zr-DFO-NCS decreased by 13.3 % (4.7 SD) (n = 3, *P* = 0.062) (Fig. 6).

## Discussion

Along with the rapidly evolving advancements in the field of cell-based therapies, there is a growing interest in improving and evaluating the efficacy of new therapies. There is a need for sensitive tools that can be used to better understand the *in vivo* migration and behaviour of transplanted cells. Long-term cell tracking with PET is gaining more interest and can serve as an efficient and important tool in the development of cell-based therapies [7, 9, 21, 30].

In this study, we focused on optimizing the synthesis of [^89^Zr]Zr-oxine and [^89^Zr]Zr-DFO-NCS and performed a direct comparison between the two tracers for the radiolabelling of hDSCs, rMac and hPBMCs. These cell types were selected for their potential in cell-based therapies and to make a comparison with previous cell labelling studies using the clinically approved radiotracer [^111^In]In-oxine [13, 19, 30].

Our optimisation of the [^89^Zr]Zr-oxine synthesis was originally inspired by the principle of hydrophilic and hydrophobic polarisation with chloroform extraction and evaporation [16, 31]. These procedures resulted in a significant decrease of RCC and unnecessary radioactive and chemical exposure, which in turn limited its increased experimental usage or up-scale production. Furthermore, any additional step in the production ordinarily decreases the RCY [32]. Sato *et al* introduced an elegant synthesis of the [^89^Zr]Zr-oxine complex in an aqueous solution that could be directly used to label the cells without the need for post-labelling purifications. However, this method utilised an additional step of buffer exchange of [^89^Zr]Zr-oxalate to zirconium chloride (^89^ZrCl_4_) using a QMA column [25]. We optimised the method by omitting the buffer exchange step, using a mixture of [^89^Zr]Zr-oxalate and oxine in a sodium acetate solution. Here, [^89^Zr]Zr-oxine was synthesised in alkaline conditions (pH 9.1) at elevated temperature (65 °C) for 60-min incubation. The advantage of our method is the simple two-step, 80-min total procedure protocol, requiring no further purification.

In terms of radiosynthesis, [^89^Zr]Zr-oxine and [^89^Zr]Zr-DFO-NCS are easily produced with high RCCs (> 95 %). However, the short shelf-life of [^89^Zr]Zr-DFO-NCS (<1.5 h), most likely due to the hydrolysis of the NCS group, greatly limits the off-site storage and research capabilities. This is not the case for [^89^Zr]Zr-oxine, where RCC and cell labelling capacity remains unchanged up to 7 days.

The previously published studies present highly inconsistent cell labelling efficiencies of both [^89^Zr]Zr-oxine and [^89^Zr]Zr-DFO-NCS, showing CLEs varying between 13 and 55 % [16, 33] and 30–72 % [14, 28], respectively. Until now, it remained unclear if these reported differences were to be attributed to different labelling methods or to different cell types. It is therefore important to conduct a head-to-head comparison to evaluate the CLE and potential effects on the same cell types using the two tracers.

Cell labelling of hDSCs, rMac and hPBMC with [^89^Zr]Zr-oxine and [^89^Zr]Zr-DFO-NCS provided a similar CLE range from 60 to 70 %. These results are comparable with previous publications using the SPECT radiotracer [^111^In]In-oxine but with significantly higher stability *in vitro* [34]. The cellular retention of [^89^Zr]Zr-oxine and [^89^Zr]Zr-DFO-NCS after 7 days in culture was 56–60 % and 26–60 %, respectively. Previous studies on [^111^In]In-oxine reported a cellular efflux of 70–75 % within 3–4 days [34, 35]. Strikingly, our results indicated a substantial superiority in cellular retention of ^89^Zr over ^111^In. One possible explanation is that ^89^Zr is a tetravalent ion while ^111^In is only trivalent, suggesting that ^89^Zr provides a more stable binding in the cytosol. In contrast, [^89^Zr]Zr-DFO-NCS relies on the abundance of free amines on the cell surface which can vary significantly between different cell types that can consequently affect the CLE of [^89^Zr]Zr-DFO-NCS. Additionally, the binding efficiency of [^89^Zr]Zr-DFO-NCS can be affected by hydrolysis and the possible presence of amines in the cell medium or residual cell fragments during cell labelling. The competition of exposed amines could therefore result in a significantly decreased CLE.

Our study also indicates that when the damaged cells had been discarded after 24 h post-labelling, the majority of the conjugated radioactivity remained bound to the viable cells for up to 7 days. Confirmed by previously published research, we propose that the ^89^Zr will most likely residualise within the cells or remain bound to the surface if the cells remain intact [14, 16, 25].

Labelling with [^89^Zr]Zr-DFO-NCS and [^89^Zr]Zr-oxine did not show any statistically significant decrease in viability in any of the cell lines. Labelling rMac with either [^89^Zr]Zr-DFO-NCS or [^89^Zr]Zr-oxine resulted in a slightly decreased phagocytotic function. Radiolabelled hDSC cells showed a significant increase in CD29, CD44, CD73 and CD105 expression for both radiotracers compared to unlabelled control cells. However, the antigen expression still corresponds to the hDSC phenotype [30, 36]. The increased expression of CD29 indicates an activation of the hDSCs [37] and CD44 expression is associated with cell differentiation and growth as well as downregulation of apoptosis [38]. Controversially, it is suggested that CD105 promotes apoptosis in rat DSC [39]. The role of CD73 in hDSCs is unclear but upregulation of CD73 in tumour and immune cells increases the adenosine production and hence ATP breakdown [40]. As such, the stress from radiolabelling of hDSCs may stimulate growth, differentiation and apoptosis. Previous studies on NK cells labelled with [^89^Zr]Zr-oxine show no significant alteration in cell functionality, phenotype or apoptotic activity at 20 kBq/10^6^ cells [41]. Another study on the radiolabelling of mesenchymal stem cells (MSC), in which the optimal level of [^89^Zr]Zr-oxine was investigated, shows no effect on the phenotype up to 1515 kBq/10^6^ cells [42]. However, a significant increase in the cell cycle arrest and apoptosis was detected. Man *et al*. demonstrated that significant DNA damage is evident in white blood cells labelled with [^89^Zr]Zr-oxine at 32.9 kBq/10^6^ cells [24]. Patrick *et al.* established that the majority of DNA damage is repaired at the 7-day time point [42]. This led us to believe that part of the cell stress could be due to significant DNA damage, explaining the increased apoptotic signal. The cell stress could also be due to the chemical exposure of the oxine/DFO-NCS to the cells, rather than due to the amount radioactivity, as earlier demonstrated with [^111^In]In-oxine [43] and later discussed as contributing to cell viability loss [44]. In the present study, we have only assessed the effects on cell viability and phenotype of the complete, radiolabelled tracers [^89^Zr]Zr-oxine and [^89^Zr]Zr-DFO-NCS, instead of unlabelled oxine or DFO-NCS by themselves. Further studies are needed to assess if the stress is temporary and reparable or permanent, in which case the level of radioactivity per cell, where possible, could be decreased to minimise the DNA damage.

## Conclusion

We optimised the synthesis procedure of [^89^Zr]Zr-oxine and [^89^Zr]Zr-DFO-NCS. Both [^89^Zr]Zr-oxine and [^89^Zr]Zr-DFO-NCS yielded similar results in terms of radiochemical conversion and cell labelling efficiency. Although the synthesis conditions for [^89^Zr]Zr-DFO-NCS were more ambient (room temperature, neutral pH), [^89^Zr]Zr-oxine appeared superior to [^89^Zr]Zr-DFO-NCS with regard to long-term stability, cellular retention, minimal variation between cell types and cell labelling efficiency after prolonged waiting times between radiosynthesis and cell labelling. Further studies are ongoing to evaluate the two radiotracers’ applicability and feasibility for long-term cell tracking *in vivo*.

## Supplementary Information

ESM 1 (PDF 105 kb)
