# Correction: Optimisation of the Synthesis and Cell Labelling Conditions for [89Zr]Zr-oxine and [89Zr]Zr-DFO-NCS: a Direct In Vitro Comparison in Cell Types with Distinct Therapeutic Applications

**DOI:** 10.1007/s11307-022-01709-1

**Published:** 2022-02-16

**Authors:** Ida Friberger, Emma Jussing, Jinming Han, Jeroen A. C. M. Goos, Jonathan Siikanen, Helen Kaipe, Mélanie Lambert, Robert A. Harris, Erik Samén, Mattias Carlsten, Staffan Holmin, Thuy A. Tran

**Affiliations:** 1 Department of Clinical Neuroscience, Karolinska Institutet, Stockholm, Sweden; 2 Department of Oncology and Pathology, Karolinska Institutet, Stockholm, Sweden; 3 Department of Radiopharmacy, Karolinska University Hospital, Stockholm, Sweden; 4 Centre for Molecular Medicine, Karolinska University Hospital, Stockholm, Sweden; 5 Department of Medical Radiation Physics and Nuclear Medicine, Karolinska University Hospital, Stockholm, Sweden; 6 Department of Laboratory Medicine, Karolinska Institutet, Stockholm, Sweden; 7 Department of Clinical Immunology and Transfusion Medicine, Karolinska University Hospital, Stockholm, Sweden; 8 Center for Cell Therapy and Allogeneic Stem Cell Transplantation (CAST), Karolinska University Hospital, Stockholm, Sweden; 9 Department of Neuroradiology, Karolinska University Hospital, Stockholm, Sweden

**Correction to: Molecular Imaging and Biology (2021) 23:952-962**

https://doi.org/10.1007/s11307-021-01622-z

This article was updated to include the correct electronic supplementary material.

