# Supplementary material for: Optimisation of the Synthesis and Cell Labelling Conditions for [89Zr]Zr-oxine and [89Zr]Zr-DFO-NCS: a Direct In Vitro Comparison in Cell Types with Distinct Therapeutic Applications
Source: Mol Imaging Biol. 2021 Jul 6;23(6):952–62. doi: 10.1007/s11307-021-01622-z (PMC8578071; doi:10.1007/s11307-021-01622-z)
Supplement: Supplementary file 1 — (PDF 105 kb) [file 11307_2021_1622_MOESM1_ESM.pdf]

Optimisation of the synthesis and cell labelling conditions for [^{89}Zr]Zr-oxine and [^{89}Zr]Zr-DFO-NCS: a direct *in vitro* comparison in cell types with distinct therapeutic applications

Ida Friberger[1], Emma Jussing[2,3], Jinming Han[1,4], Jeroen A.C.M. Goos[1,2,3], Jonathan Siikanen[2,5], Helen Kaipe[6,7], Mélanie Lambert[8], Robert A. Harris[1,4], Erik Samén[2,3], Mattias Carlsten[8,9], Staffan Holmin[1,10], Thuy A. Tran*[1,2,3]

[1]Department of Clinical Neuroscience, Karolinska Institutet, Stockholm, Sweden.
[2]Department of Oncology and Pathology, Karolinska Institutet, Stockholm, Sweden.
[3]Department of Radiopharmacy, Karolinska University Hospital, Stockholm, Sweden.
[4]Centre for Molecular Medicine, Karolinska University Hospital, Stockholm, Sweden.
[5]Department of Medical Radiation Physics and Nuclear Medicine, Karolinska University Hospital, Stockholm, Sweden.
[6]Department of Laboratory Medicine, Karolinska Institutet
[7]Dept of Clinical Immunology and Transfusion Medicine, Karolinska University Hospital, Stockholm, Sweden
[8]Department of Medicine in Huddinge, Karolinska Institutet, Stockholm, Sweden.
[9]Center for Cell Therapy and Allogeneic Stem Cell Transplantation (CAST), Karolinska University Hospital, Stockholm, Sweden.
[10]Department of Neuroradiology, Karolinska University Hospital, Stockholm, Sweden.

*Corresponding author: Thuy Tran, PhD.

Thuy.tran@ki.se

MATERIALS AND METHODS

Production of Zirconium-89

^{89}Zr was produced in-house with a cyclotron (PETtrace 800, GE Healthcare) using the $^{89}Y(p,n)^{89}Zr$ reaction. Natural (monoisotopic) yttrium foils of 15.5 mm diameter, 0.25 mm thick were pneumatically transferred to a solid target system (EDS/PTS, Comecer) and irradiated with 25 µA protons in a cyclotron for 1-3 hrs. Proton energy was degraded to a nominal 12.6 MeV to minimize co-production of long-lived ^{88}Zr and ^{88}Y. Separation of ^{89}Zr from ^{89}Y was performed with a separation module (Taddeo PRF, Comecer) using a home-made hydroxamate column as described previously [1, 2]. Prior to separation, the column was activated with acetonitrile (TraceSELECT, Honeywell™ Riedel-de-Haën™), H_2O (TraceSELECT, Honeywell Riedel-de Haen) and 2 M hydrochloric acid (HCl). The irradiated yttrium foil was dissolved with 6 M HCl (Fisher Scientific) and diluted with H_2O. The ^{89}Zr solution was loaded onto the resin column and washed with 2 M HCl and H_2O. ^{89}Zr was then eluted with 1.6 mL, 1 M oxalic acid (Sigma-Aldrich). The experimental saturation yield of [^{89}Zr]Zr-oxalate was 1440 ± 450 MBq/µA (n = 11). The radionuclidic purity (RNP) of ^{89}Zr-eluates was determined by gamma spectroscopy using an energy and efficiency calibrated High Purity Germanium detector (Canberra with Cryo-Cycle II Hybrid Cryostat). RNP of ^{89}Zr was over 99.99%.

Optimization of [^{89}Zr]Zr-oxine synthesis

To exclude the need for chloroform, different non-toxic buffers and solvents were investigated, avoiding the need for separation and evaporation. To accelerate the reaction, the concentration was increased by decreasing the volume and the temperature was increased (not exceeding the melting point of oxine at 75°C). To investigate the optimal incubation and pH conditions, a pH range from 5.0 to 10.0 was tested, following escalating incubation times of 10, 20, 30, 40, 50 and 60 minutes. The final protocol was obtained: oxine (Honeywell) was dissolved in 99% ethanol (Sigma-Aldrich) to a concentration of 204 mM and incubated on a heat shaker (Eppendorf Thermomixer C) at 65°C for approximately 5 minutes.

Aliquots of 5-20 MBq [^{89}Zr]Zr-oxalate were mixed with 50 µL 0.1 M sodium acetate buffer (NaOAc, pH 2.5-3.0) (Merck Millipore) in a centrifuge tube. Thereafter, 0.1 mg dissolved oxine was added to the NaOAc mixture, 7.8 mM (1.1 SD, where SD = standard deviation) and the pH was adjusted to 9.1 using 1 M sodium carbonate (Honeywell). The sample was incubated for

60 minutes at 65°C. The shelf-life of [^{89}Zr]Zr-oxine was tested for complex stability and cell labelling capacity after 7 days. The RCC was determined by instant thin-layer chromatography (iTLC). Approximately 1-2 µL of the sample was taken and spotted onto an iTLC strip of SG-silica gel (Agilent Technologies), eluted in a mobile phase of 20 mM citrate solution (Fluka, Sigma-Aldrich), pH 4, analysed with a radio-TLC scanner (AR-2000, Eckert & Ziegler) and quantified using WinScan software version 3.0. The unbound ^{89}Zr travels with the hydrophilic solvent front (retardation factor, Rf ~0.9) while the hydrophobic [^{89}Zr]Zr-oxine remains at the origin (Rf ~ 0). The RCC was also confirmed by radio high-performance liquid chromatography (radio-HPLC) (as described below). RCC was consistently over 95%, therefore no further separation was needed.

[^{89}Zr]Zr-DFO-NCS synthesis

To achieve a stable RCC of >95% without the need for purification, we optimised several conditions such as synthesis buffer, reaction time, DFO-NCS concentration, and temperature as below. The RCC in all tests was determined by iTLC using a mobile phase of 50 mM DTPA (Sigma-Aldrich) pH 4). [^{89}Zr]Zr-DFO-NCS remained at the origin (Rf ~ 0) while unbound ^{89}Zr was chelated by DTPA, travelling with the solvent front (Rf ~ 0.9). The RCC was also confirmed by radio-HPLC (as described below).

Synthesis buffer and reaction time: In the initial protocol, 12 µg (30 µM) DFO-NCS (Macrocyclics) dissolved in DMSO (Sigma-Aldrich), was added to a buffer solution of pH 7.4, containing 250 µL HEPES buffer (Gibco, Thermo Fisher), 200 µL Tris buffer (Mallinkrodt), and 50-100 µL [^{89}Zr]Zr-oxalate. The [^{89}Zr]Zr-DFO-NCS solution was set to pH 7.0-7.4 using 1 M sodium carbonate and incubated at room temperature. To further simplify the protocol, the synthesis was performed as described but using PBS buffer (Sigma Aldrich) instead of a combination of HEPES/Tris buffer. To assess the optimal reaction time, an aliquot of the labelling solution was withdrawn at different reaction time points, from 10 – 60 minutes and the RCC was assessed by iTLC analysis (see Figure 4B in the results section).

DFO-NCS concentration: Different DFO-NCS concentrations were labelled and the RCC was determined over time up to 60 minutes. By decreasing the volume and increasing the amount of DFO-NCS, the concentrations of DFO-NCS of 27, 53, 80 and 106 µM were tested (see Figure 3E for results).

After all these optimisations, a final protocol was obtained: DFO-NCS (p-SCN-Bn-Deferoxamine) was dissolved in DMSO to a final concentration of 5.3 mM. Approximately 5-

25 MBq of [^{89}Zr]Zr-oxalate was suspended in 50 µL 0.5 M PBS buffer (Sigma-Aldrich) and the pH was adjusted to 7.0-7.4 using 1 M sodium carbonate. Next, 1 µL of dissolved DFO-NCS (4 µg) was added to the [^{89}Zr]Zr-oxalate mixture to achieve final molarity of approximately 70 µM. Incubation was performed under mild agitation (300 rpm) for 60 minutes at room temperature. The [^{89}Zr]Zr-DFO-NCS complex is known to be unstable in aqueous conditions, so the shelf-life of [^{89}Zr]Zr-DFO-NCS was tested and cell labelling capacity up to 24 hrs.

Radio-HPLC analysis of [^{89}Zr]Zr-oxine and [^{89}Zr]Zr-DFO-NCS

To confirm the RCC, analysis using radio high-performance liquid chromatography (radio-HPLC) was performed. An HPLC system (UFLC Shimadzu), equipped with a radio detector (Bioscan, Flow-Count), was used. An Atlantis C18AX MPFIT column (250x4.6 mm 5 µm; Waters, Solna, Sweden) was used with a gradient of acetonitrile (Thermo Fisher)/water/trifluoroacetic acid (Sigma-Aldrich) from 0/100/0.1 to 100/0/0.1 (v/v/v) for 30 minutes at a flow rate of 1 mL/min and wavelength 254 nm, analysed with LabSolutions Chromatography software (Shimadzu).

Cell preparations

Human decidual stromal cells

The human decidual stromal cells (hDSCs) were isolated, prepared and cultured as previously described [3, 4]. Briefly, hDSCs were isolated from human placenta, obtained through a caesarean section of healthy donors after informed consent, according to legislation by the Institutional Ethical Review Board (2009/418-31/4, 2010/2061-32, Dnr: 2015/1848-31/2). The recovered placenta was washed in PBS to remove blood and was then dissected to isolate the desired maternal decidual parietalis layer from the fetal membrane. The decidua was cut into 2-3 cm^2 pieces and cultured in T175 flasks in complete DMEM medium, containing 10% FBS and 1% penicillin-streptomycin (Sigma-Aldrich). When colonies reached 95% confluence, the hDSCs were resuspended with trypsin 0.05% containing 0.2 g/L ethylenediaminetetraacetic acid (EDTA) (Hyclone), then washed and re-cultured in complete DMEM medium. At passage 3 or 4, the cells were gradually frozen in 10% dimethyl sulfoxide (DMSO) (Sigma-Aldrich). The hDSCs were analysed using microsatellite polymorphism (with capillary electrophoresis) and tested positive for antigen expression of HLA class I (W6/32) (DAKO), CD29 (MAR4), CD44 (G44-26), CD73 (AD2), CD105 (266) (BD) and negative for HLA class II (CR3/43) (DAKO), CD14 (MϕP9), CD31 (WM59), CD34 (8G12) and CD45 (H130) (BD) by flow

cytometry, hence confirming that the cells were of maternal origin and of mesenchymal lineage [4, 5].

Rat bone marrow-derived macrophages

In accordance with the ethics approval (9328-2019 and N138/14), rat bone marrow-derived macrophages (rMac) were collected as previously described [6]. Briefly, the femurs were surgically removed and the bones were cut at both ends to expose the bone marrow (BM) cells. The BM cells were then thoroughly flushed with Dulbecco's Modified Eagle's Medium (DMEM) (Sigma-Aldrich) using a syringe and needle. To disaggregate potential cell clusters, the cells were carefully resuspended and passed through a cell strainer (40 μm, BD Biosciences). The collected cell mixture was then suspended in complete DMEM containing 20% fetal bovine serum (Sigma-Aldrich), penicillin-streptomycin (Sigma-Aldrich), L-glutamine (Sigma-Aldrich), 2-mercaptoethanol (Gibco, Life Technologies) and sodium pyruvate (Thermo Fisher) and was then stimulated with 20 ng/mL rat macrophage colony-stimulating factor M-CSF (400-28, PeproTech). Cells were then cultured in T175 culture flasks for 8 days in a humid incubation chamber at 37°C and with 5% CO_2. Mature macrophages were resuspended in PBS and characterised using flow cytometric analysis (BD Biosciences or Merck Guava H12), as previously described [6]. Cell proliferation was determined using dimethyl sulfoxide with 5 mM carboxyfluorescein succinimidyl ester (CFSE) (BioLegend), measured by flow cytometry and analysed using the Calibur software (FlowJo v10, BD Biosciences).

Human peripheral blood mononuclear cells

Human peripheral blood mononuclear cells (hPBMC) were isolated from buffy coats under ethics approval Dnr 2006/229-31/1 as previously described [7]. In short, blood cells were separated with Ficoll Paque (Pharmacia Biotech AB) gradient centrifugation. The hPBMCs were frozen in 90% fetal calf serum (Gibco, Life Technologies) and 10% DMSO and stored in liquid nitrogen until use. When thawed, cells were kept in complete Roswell Park Memorial Institute medium 1640 (RPMI 1640) media containing 10% FBS and 1% penicillin-streptomycin until cell labelling.

Labelling cells with [^{89}Zr]Zr-oxine

The hDSCs, rMac and hPBMC were labelled with [^{89}Zr]Zr-oxine by adding 0.25 mL 0.5 M PBS to the [^{89}Zr]Zr-oxine solution, followed by neutralization using 1 M oxalic acid and the addition of 0.05 mL Tris buffer (Mallinckrodt). [^{89}Zr]Zr-oxine (5-15 MBq, 0.23 µmol/10^6 cells (0.19 SD) was added to 1.5-10x10^6 cells in 0.70 mL 0.5 M PBS solution, giving a final volume

of 1.1 mL. Cells were incubated for 40 minutes at room temperature or 37°C in an incubator, and they were gently vortexed every 10 minutes to prevent aggregation. Cells were centrifuged (5 minutes, 1000 rpm) and washed twice with 0.5 M PBS to remove unbound [^{89}Zr]Zr-oxine. Cell labelling efficiency, CLE was determined by measuring the fractions of radioactivity left in the cell pellet after repeated centrifugation and wash. Cell viability for both labelled and unlabelled control cells was determined with Trypan Blue staining and counted with a cell counter (Countess II Automated Cell Counters, Invitrogen). Evaluation of ^{89}Zr retention, the phagocytic ability of rMac and phenotype of hDSCs was performed using flow cytometry (as described below). All cell lines were also radiolabelled with 5-15 MBq unbound ^{89}Zr as a negative control for [^{89}Zr]Zr-oxine cell labelling.

Labelling cells with [^{89}Zr]Zr-DFO-NCS

hDSCs, rMac and hPBMC were suspended in 1 mL 0.5 M PBS (2-10x10^6) at pH 7.4. A neutralised solution of [^{89}Zr]Zr-DFO-NCS was added to the cells (7.5 µM (2.3 SD)) in a total volume of 1-1.2 mL. Subsequently, cells were incubated for 40 minutes at room temperature under gentle vortexing every 10 minutes to prevent aggregation. Cells were then centrifuged (5 minutes, 1000 rpm) and washed twice with 0.5 M PBS to remove free [^{89}Zr]Zr-DFO-NCS. A toxicity threshold test of [^{89}Zr]Zr-DFO-NCS was performed on hDSC with DFO-NCS concentrations of 6, 9, 13, 16 or 19 µM. Cell labelling efficiency, CLE was determined by measuring the fractions of radioactivity left in the cell pellet after repeated centrifugation and wash. All cell lines were also radiolabelled with 5-15 MBq unbound ^{89}Zr as a negative control for [^{89}Zr]Zr-DFO-NCS cell labelling. Cell viability for both labelled and unlabelled cells was determined with Trypan Blue staining and counted with a cell counter. Following, ^{89}Zr retention measurements, determination of the phagocytic ability of rMac and phenotype of hDSCs were performed using flow cytometry.

References used in the supplementary section

1. Holland JP, Sheh Y, Lewis JS (2009) Standardized methods for the production of high specific-activity zirconium-89. Nucl Med Biol 36:729–739. https://doi.org/10.1016/j.nucmedbio.2009.05.007

2. Verel I, Visser GWM, Boellaard R, et al (2003) 89Zr immuno-PET: comprehensive procedures for the production of 89Zr-labeled monoclonal antibodies. J Nucl Med Off Publ Soc Nucl Med 44:1271–1281

3. Erkers T, Nava S, Yosef J, et al (2013) Decidual stromal cells promote regulatory T cells and suppress alloreactivity in a cell contact-dependent manner. Stem Cells Dev 22:2596–2605. https://doi.org/10.1089/scd.2013.0079

4. Arnberg F, Lundberg J, Olsson A, et al (2016) Intra-arterial Administration of Placenta-Derived Decidual Stromal Cells to the Superior Mesenteric Artery in the Rabbit: Distribution of Cells, Feasibility, and Safety. Cell Transplant 25:401–410. https://doi.org/10.3727/096368915X688191

5. Ringdén O, Erkers T, Nava S, et al (2013) Fetal membrane cells for treatment of steroid-refractory acute graft-versus-host disease. Stem Cells Dayt Ohio 31:592–601. https://doi.org/10.1002/stem.1314

6. Weischenfeldt J, Porse B (2008) Bone Marrow-Derived Macrophages (BMM): Isolation and Applications. CSH Protoc 2008:pdb.prot5080

7. Hanson MGV, Ozenci V, Carlsten MCV, et al (2007) A short-term dietary supplementation with high doses of vitamin E increases NK cell cytolytic activity in advanced colorectal cancer patients. Cancer Immunol Immunother CII 56:973–984. https://doi.org/10.1007/s00262-006-0261-4
